# School District Crisis Preparedness, Response, and Recovery Plans — United States, 2006, 2012, and 2016

**DOI:** 10.15585/mmwr.mm6730a1

**Published:** 2018-08-03

**Authors:** Judy Kruger, Nancy Brener, Rebecca Leeb, Amy Wolkin, Rachel Nonkin Avchen, Eric Dziuban

**Affiliations:** ^1^Division of State and Local Readiness, Office of Preparedness and Response, CDC; ^2^Division of Adolescent and School Health, National Center for HIV/AIDS, Viral Hepatitis, STD, and TB Prevention, CDC; ^3^Division of Human Development and Disability, National Center on Birth Defects and Developmental Disabilities, CDC; ^4^Office of Science and Public Health Practice, Office of Public Health Preparedness and Response, CDC.

Children spend the majority of their time at school and are particularly vulnerable to the negative emotional and behavioral impacts of disasters, including anxiety, depressive symptoms, impaired social relationships, and poor school performance (*1*). Because of concerns about inadequate school-based emergency planning to address the unique needs of children and the adults who support them, Healthy People 2020 includes objectives to improve school preparedness, response, and recovery plans (Preparedness [PREP]-5) (*2*). To examine improvements over time and gaps in school preparedness plans, data from the 2006, 2012, and 2016 School Health Policies and Practices Study (SHPPS) were analyzed to assess changes in the percentage of districts meeting PREP-5 objectives. Findings from these analyses indicate that districts met the PREP-5 objective for requiring schools to include post-disaster mental health services in their crisis preparedness plans for the first time in 2016. However, trend analyses did not reveal statistically significant increases from 2006 to 2016 in the percentage of districts meeting any of the PREP-5 objectives. Differences in preparedness were detected in analyses stratified by urbanicity and census region, highlighting strengths and challenges in emergency planning for schools. To promote the health and safety of faculty, staff members, children, and families, school districts are encouraged to adopt and implement policies to improve school crisis preparedness, response, and recovery plans.

SHPPS is a national survey periodically conducted by CDC to assess school health policies and practices (*3*). This report used district-level data from the 2006, 2012, and 2016 surveys. In each study year, a nationally representative sample of public school districts is drawn using a two-stage sample design. Five to seven questionnaires, each assessing a different component of school health, are administered in each sampled district via paper and pencil or online. This report summarizes results from the crisis preparedness module within the healthy and safe school environment questionnaire. Across the three study years, the number of sampled districts that completed this questionnaire ranged from 461 to 697, and the response rates ranged from 64.0% to 66.5%.

Each district identified the respondent who had primary responsibility for, or was most knowledgeable about, the content of each questionnaire. Respondents to the crisis preparedness module were asked whether their school district required schools to have a comprehensive plan to address crisis preparedness, response, and recovery that included four specific topics identified in PREP-5: family reunification procedures, procedures for responding to pandemic influenza or other infectious disease outbreaks (only asked in 2012 and 2016), provisions for students and staff members with special needs, and provision of mental health services for students and staff members after a crisis. Respondents also were asked whether their district provided funding for training or offered training on their crisis preparedness plans to school faculty and staff members, students, and students’ families, and whether their district offered education on crisis preparedness, response, and recovery to students’ families. To categorize SHPPS school districts accurately into U.S. Census regions, SHPPS data were linked to extant data from the Market Data Retrieval database (*4*), a commercial database that compiles a list of K–12 schools in the United States along with their characteristics. Analyses were stratified by census region (Midwest, Northeast, South, or West); urbanicity (city, suburb, town, or rural); and district enrollment size (small [≤4,999 students], medium [5,000–9,999], or large [≥10,000]).

Data from each study year were weighted to provide national estimates. Analyses using statistical software accounted for the complex sample design. Prevalence estimates and 95% confidence intervals were computed for all point estimates. Statistical significance (p<0.05) for linear trends was determined using logistic regression analyses with data from all 3 years. The 2016 data only were used for descriptive statistics related to training and education. T-tests were used to determine differences between subgroups, with p-values <0.05 considered statistically significant.

Overall, no significant changes over time were detected in the percentage of districts that required schools to include specific topics in their school crisis preparedness, response, and recovery plans that correspond to the Healthy People 2020 PREP-5 objectives (Table 1) (Table 2). Notably, the Healthy People 2020 district requirements for school plans to include provision of mental health services for students, faculty, and staff members after a crisis (PREP-5.4; target ≥76.2%) was met (77.6%) for the first time in 2016 (Table 2).

**TABLE 1:** Percentage of districts that require schools to include family reunification or infectious disease outbreak in their school crisis preparedness, response, and recovery plans by selected characteristics — School Health Policies and Practices Study, United States, 2006, 2012, 2016

District characteristic	Year, % (95% CI)			
	2006	2012	2016	P-value
Topic: family reunification procedures* (PREP^†^ 5.1 target = 74.6%)				

No. of observations	402	599	559	—
**Percentage of districts**	**65.3 (59.5–70.6)**	**67.8 (63.8–71.5)**	**74.4 (70.5–77.9)**	**0.068**
**Urbanicity**				
City	64.0 (34.6–85.6)	87.7 (76.4–94.0)	85.6 (69.8–93.9)	0.079
Suburb	70.8 (59.7–79.8)	75.4 (68.4–81.3)	82.5 (74.6–88.4)	0.029^§^
Town	68.5 (52.6, 81.0)	63.0 (52.5, 72.4)	77.4 (67.7–84.8)	0.392
Rural	63.3 (55.8–70.2)	61.0 (54.8–66.8)	65.8 (59.8–71.3)	0.673
**District enrollment size (no. of students)**				
Small (≤4,999)	64.0 (57.8–69.7)	65.3 (60.9–69.5)	71.6 (67.2–75.6)	0.130
Medium (5,000–9,999)	77.9 (57.4–90.2)	83.8 (70.5–91.8)	87.7 (73.3–94.9)	0.268
Large (≥10,000)	76.6 (52.0–90.8)	83.2 (68.6–91.8)	89.9 (77.8–95.8)	0.107
**U.S. Census region** ^¶^				
Midwest	57.7 (48.5–66.3)	60.2 (53.4 – 66.7)	69.4 (62.8–75.3)	0.062
Northeast	61.7 (46.1–75.2)	72.0 (62.9–79.6)	77.4 (68.6–84.3)	0.050**
South	72.8 (62.5–81.1)	71.6 (64.3–78.0)	81.1 (74.2–86.5)	0.248
West	74.3 (58.8–85.4)	73.6 (63.0–82.1)	72.3 (61.0–81.4)	0.836
**Topic: procedures for responding to pandemic influenza or other infectious disease outbreak^††^ (PREP^†^ 5.2 target = 75.9%)**				
**No. of observations**	**404**	**595**	**560**	**—**
**Percentage of districts**	**—**	**69.0 (65.0–72.7)**	**65.3 (61.2–69.3)**	**0.359**
**Urbanicity**				
City	—	80.6 (67.7–89.2)	72.6 (55.0–85.2)	0.423
Suburb	—	70.8 (63.5–77.2)	75.8 (67.2–82.7)	0.237
Town	—	70.4 (60.0–79.0)	69.4 (59.2–78.1)	0.870
Rural	—	65.3 (59.1–70.9)	56.2 (50.0–62.1)	0.012^§^
**District enrollment size (no. of students)**				
Small (≤4,999)	—	66.7 (62.3–70.8)	62.9 (58.3–67.3)	0.321
Medium (5,000–9,999)	—	84.0 (69.9–92.3)	73.5 (57.6–85.0)	0.219
Large (≥10,000)	—	83.4 (69.1–91.8)	83.4 (69.7–91.6)	0.994
**U.S. Census region** ^¶^				
Midwest	—	57.9 (51.1–64.5)	57.3 (50.3–63.9)	0.904
Northeast	—	75.2 (66.3–82.4)	70.9 (62.0–78.5)	0.403
South	—	79.4 (72.4–85.0)	69.9 (62.0–76.8)	0.053**
West	—	68.5 (57.5–77.7)	69.2 (57.4–79.0)	0.935

* Adopted a policy requiring schools’ crisis plans to include family reunification procedures.

^†^ Healthy People 2020 Preparedness (PREP) objective 5.

^§^ Statistically significant (p<0.05).

^¶^ Regions: *Northeast*: Connecticut, Maine, Massachusetts, New Jersey, New Hampshire, New York, Pennsylvania, Rhode Island, and Vermont; *Midwest*: Illinois, Indiana, Iowa, Kansas, Michigan, Minnesota, Missouri, Nebraska, North Dakota, Ohio, South Dakota, and Wisconsin; *South*: Alabama, Arkansas, Delaware, District of Columbia, Florida, Georgia, Kentucky, Louisiana, Maryland, Mississippi, North Carolina, Oklahoma, South Carolina, Tennessee, Texas, Virginia and West Virginia; *West*: Alaska, Arizona, California, Colorado, Hawaii, Idaho, Montana, Nevada, New Mexico, Oregon, Utah, Washington, and Wyoming.

** p = 0.05.

^††^ Adopted a policy requiring schools’ crisis plans to include procedures for responding to pandemic influenza or other infectious disease outbreak. Question was not asked in 2006.

**TABLE 2:** Percentage of districts that require schools to include provisions for special needs or mental health services in their school crisis preparedness, response, and recovery plans by selected characteristics — School Health Policies and Practices Study, United States, 2006, 2012, 2016

District characteristic	Year, % (95% CI)			P-value
	2006	2012	2016	
**Topic: provisions for students and staff members with special needs^¶^ (PREP^†^ 5.3 target = 87.9%)**				
**No. of observations**	**404**	**596**	**561**	**—**
**Percentage of districts**	**77.4 (72.1–82.0)**	**79.9 (76.3–83.0)**	**79.8 (76.2–83.0)**	**0.538**
**Urbanicity**				
City	91.2 (68.8–98.0)	88.7 (77.1, 94.8)	84.9 (69.7–93.2)	0.511
Suburb	84.6 (74.8–91.1)	85.0 (78.8–89.6)	90.6 (84.0–94.7)	0.150
Town	79.2 (63.1–89.5)	82.1 (72.6–88.8)	82.1 (72.8–88.7)	0.663
Rural	74.8 (67.7–80.7)	74.5 (68.7–79.6)	71.6 (65.7–76.8)	0.460
**District enrollment size (no. of students)**				
Small (≤4,999)	76.5 (70.7–81.4)	78.5 (74.5–82.0)	77.8 (73.7–81.4)	0.727
Medium (5,000–9,999)	88.2 (74.2–95.1)	87.2 (74.1–94.2)	90.3 (76.3–96.4)	0.778
Large (≥10,000)	82.8 (54.6–95.1)	90.8 (78.0–96.5)	90.7 (79.1–96.1)	0.353
**U.S. Census region** ^§^				
Midwest	72.4 (63.5–79.8)	72.2 (65.6–78.0)	75.8 (69.6–81.1)	0.571
Northeast	78.6 (63.1–88.8)	87.6 (80.0–92.5)	87.1 (80.0–91.9)	0.211
South	81.5 (71.8–88.4)	87.8 (81.8–92.0)	86.2 (79.8–90.8)	0.272
West	82.6 (67.9–91.4)	73.0 (62.3–81.6)	71.7 (60.1–81.0)	0.228
**Topic: provision of mental health services for students, faculty, and staff members after a crisis occurred^¶^ (PREP^†^ 5.4 target = 76.2%)**				
**No. of observations**	**404**	**595**	**560**	**—**
**Percentage of districts**	**73.0 (67.4–77.9)**	**69.3 (65.4–73.0)**	**77.6 (73.9–80.9)**	**0.424**
**Urbanicity**				
City	91.3 (68.8–98.1)	84.1 (72.2–91.5)	81.6 (63.9–91.7)	0.343
Suburb	84.0 (75.3–90.1)	75.2 (68.1–81.1)	87.2 (79.5–92.2)	0.632
Town	70.5 (53.9–83.0)	65.7 (55.1–74.9)	83.2 (74.6–89.3)	0.169
Rural	70.1 (62.8–76.5)	63.9 (57.8–69.7)	68.7 (62.8–74.1)	0.669
**District enrollment size (no. of students)**				
Small (≤4,999)	70.9 (64.8–76.3)	67.7 (63.4–71.8)	75.8 (71.6–79.5)	0.405
Medium (5,000–9,999)	90.8 (78.0–96.5)	75.3 (61.1–85.6)	88.0 (73.9–95.0)	0.566
Large (≥10,000)	93.3 (76.3–98.4)	83.7 (69.6–92.0)	86.1 (70.8–94.0)	0.294
**U.S. Census region** ^§^				
Midwest	70.6 (61.7–78.2)	60.1 (53.3–66.6)	74.2 (67.9–79.6)	0.753
Northeast	72.6 (56.8–84.2)	80.4 (71.9–86.7)	85.8 (78.4–90.9)	0.046**
South	74.1 (63.8–82.3)	72.7 (65.3–78.9)	79.2 (71.7–85.1)	0.493
West	79.2 (63.3–89.4)	71.6 (60.8–80.3)	73.2 (61.7–82.2)	0.462

* Adopted a policy requiring schools’ crisis plans to include family reunification procedures.

^†^ Healthy People 2020 Preparedness (PREP) objective 5.

^§^ Regions: *Northeast*: Connecticut, Maine, Massachusetts, New Jersey, New Hampshire, New York, Pennsylvania, Rhode Island, and Vermont; *Midwest*: Illinois, Indiana, Iowa, Kansas, Michigan, Minnesota, Missouri, Nebraska, North Dakota, Ohio, South Dakota, and Wisconsin; *South*: Alabama, Arkansas, Delaware, District of Columbia, Florida, Georgia, Kentucky, Louisiana, Maryland, Mississippi, North Carolina, Oklahoma, South Carolina, Tennessee, Texas, Virginia and West Virginia; *West*: Alaska, Arizona, California, Colorado, Hawaii, Idaho, Montana, Nevada, New Mexico, Oregon, Utah, Washington, and Wyoming.

^¶^ Adopted a policy requiring schools’ crisis plans to include provision of mental health services for students, faculty, and staff members after a crisis occurred.

** Statistically significant (p< 0.05).

Assessing district requirements by subgroup identified a significant increase in the percentage of districts in suburban areas that required schools to include family reunification procedures in their plans (PREP-5.1) from 2006 to 2016 and a linear increase in this requirement among districts in the Northeast (p = 0.05) (Table 1). The percentage of school districts that required schools to include procedures for responding to pandemic influenza or other infectious disease outbreaks in their plans (PREP-5.2) decreased significantly in rural areas (p<0.05) and among districts in the South (p = 0.05) (Table 1). By 2016, all Healthy People 2020 targets assessed were met in large school districts, although trends were not statistically significant.

In 2016, large districts were significantly more likely than were small districts to provide funding for or offer training on crisis preparedness for school faculty, staff members, and students’ families (p<0.05) (Table 3). Compared with districts in the Midwest, districts in the South were less likely to provide funding for training or offer training on crisis preparedness for school faculty and staff members (p<0.05). In contrast, districts in the Midwest were less likely than were those in the Northeast, South, and West to provide funding for training or to offer training on crisis preparedness for students’ families (p<0.05). Districts in the Midwest also were less likely than were those in the West to offer education on crisis preparedness, response, and recovery to students’ families (p<0.05).

**TABLE 3:** Percentage of districts that provided funding for training or offered training on crisis preparedness, by district-level characteristics — School Health Policies and Practices Study (SHPPS), United States, 2016

Group offered training	Provided funding for training or offered training on crisis preparedness,* % (95% CI)			Offered education to students’ families,^†^ % (95% CI)
	School faculty and staff members	Students	Students’ families	
**No. of observations**	**543**	**537**	**539**	**558**
**Percentage of districts**	**89.6 (86.4–92.0)**	**59.5 (55.0–63.7)**	**17.6 (14.2–21.0)**	**21.6 (18.2–25.5)**
**District characteristic**				
**Urbanicity**				
City	88.4 (70.3–96.0)	58.6 (42.0–73.5)	23.2 (12.2–39.6)	20.9 (10.8–36.7)
Suburb	92.5 (86.1–96.1)	61.4 (51.9–70.1)	18.8 (12.4–27.3)	21.1 (14.3–29.8)
Town	86.4 (76.7–92.5)	58.2 (47.5–68.2)	17.6 (11.2–26.5)	20.1 (12.7–30.3)
Rural	89.2 (84.7–92.5)	59.3 (53.0–65.2)	15.4 (11.2–20.7)	21.8 (17.1–27.3)
**District enrollment size (no. of students)**				
Small (≤4,999)	88.4^§^ (84.8–91.3)	58.0 (53.1–62.6)	15.0^§^ (11.9–18.8)	20.5 (16.9–24.7)
Medium (5,000–9,999)	92.6 (79.0–97.6)	65.4 (48.9–78.9)	20.4 (10.7–35.5)	21.8 (11.3–38.0)
Large (≥10,000)	97.4 (88.3–99.5)	68.5 (51.5–81.6)	37.9 (23.4–55.0)	32.9 (19.4–50.0)
**U.S. Census region** ^¶^				
Midwest	93.5** (89.3–96.1)	63.0 (56.1–69.5)	8.4**^,††,§§^ (5.2–13.1)	16.0^§§^ (11.7–21.6)
Northeast	88.6 (80.1–93.8)	55.0 (45.3–64.4)	20.9 (14.1–29.7)	21.1 (14.2–30.0)
South	86.7 (80.0–91.3)	57.2 (48.8–65.2)	20.3 (14.4–27.9)	23.7 (17.3–31.4)
West	86.3 (75.4–92.9)	60.1 (47.5–71.4)	28.3 (18.5–40.7)	30.5 (20.8–42.4)
**Total**	**89.6 (86.4–92.0)**	**59.5 (55.0–63.7)**	**17.6 (14.2–21.0)**	**21.6 (18.2–25.5)**

* Districts that responded “yes” to the question “During the past two years, has your district provided funding for or offered training on the crisis preparedness, response and recovery plan to…a) school faculty and staff members, b) students, c) students’ families?”

^†^ Districts that responded “yes” to the question “During the past two years, has your district offered education on crisis preparedness, response, and recovery to students’ families?”

^§^ Significant difference (p<0.05) between districts with small and large enrollment size.

^¶^ Regions: *Northeast*: Connecticut, Maine, Massachusetts, New Jersey, New Hampshire, New York, Pennsylvania, Rhode Island, and Vermont; *Midwest*: Illinois, Indiana, Iowa, Kansas, Michigan, Minnesota, Missouri, Nebraska, North Dakota, Ohio, South Dakota, and Wisconsin; *South*: Alabama, Arkansas, Delaware, District of Columbia, Florida, Georgia, Kentucky, Louisiana, Maryland, Mississippi, North Carolina, Oklahoma, South Carolina, Tennessee, Texas, Virginia and West Virginia; *West*: Alaska, Arizona, California, Colorado, Hawaii, Idaho, Montana, Nevada, New Mexico, Oregon, Utah, Washington, and Wyoming.

** Significant difference (p<0.05) between Midwest and South districts.

^††^ Significant difference (p<0.05) between Midwest and Northeast districts.

^§§^ Significant difference (p<0.05) between Midwest and West districts.

## Discussion

These findings highlight strengths and challenges in emergency planning for schools. In 2016, the Healthy People 2020 goal requiring school districts to have plans in place that include provision of mental health services for students, faculty, and staff members after a crisis was achieved nationally for the first time, suggesting that school districts increasingly recognize the importance of addressing post-disaster mental health needs as a vital part of crisis recovery. In addition, over the past decade, improvements were made for inclusion of family reunification procedures after a crisis at the national level, particularly in suburban schools and schools in the northeastern United States (*5*).

Despite this progress, gaps in achieving school preparedness goals at the national level persist, and progress in many essential areas is minimal. Whereas the majority of school districts have plans to address mental health needs and family reunification after an emergency, nationally, approximately one in four districts fall short of these goals, and one in three school districts does not have policies in place to prepare for an infectious disease outbreak. Because schools often function as community hubs, these gaps in preparedness planning leave communities potentially vulnerable to critical public health threats.

Preparedness planning was not consistent across localities. The percentage of rural school districts that included procedures for responding to pandemic influenza or other infectious disease outbreaks in their preparedness plans decreased significantly over time and was lower than the percentage among urban and suburban districts and towns. Furthermore, compared with large districts, a significantly lower percentage of small districts provided funding for training or offered training for crisis preparedness for school faculty, staff members, and students’ families. Because schools can be a central gathering place during an emergency in low population density areas, the decreases in infectious disease preparedness plans and lack of resources to support emergency preparedness could lead to a gap in coverage when an event occurs. School administrators have the opportunity to lead health promotion and safety in rural and smaller communities. Schools can serve as a centralized, familiar rallying point for communities during crises; however, technical support and resources are needed to ensure successful planning for administrators. Regular training regarding crisis preparedness, response, and recovery for students and their families is essential to ensuring that communities are ready when disaster strikes. School districts can partner with local and regional public health departments to determine how best to use limited resources, identify emerging themes in responses, and review emergency operations plans to identify best practices.

To promote the health and safety of faculty, staff members, children, and families and meet the Healthy People 2020 preparedness targets, more school districts can adopt and implement preparedness policies. Adoption of family reunification procedures might include steps to determine alternative school sheltering locations and family communication messaging (e.g., text messaging) to allow schools and communities to avoid extensive challenges to reuniting families, such as those observed after Hurricane Katrina (*6*,*7*). Timely family reunification promotes post-disaster recovery for adults and children, benefitting the health of communities and the population as a whole (*7*). Strengthening policies and planning for infectious disease outbreaks is vital to ensuring that communities remain healthy and productive (*8*). The 2014 Ebola outbreak in West Africa closed schools in affected areas for up to 10 months (*9*), compromising the health and well-being of children, staff members, and faculty who rely on schools for a sense of normalcy during a crisis. Therefore, school districts should consider developing customized protocols in the event of an outbreak of seasonal influenza (*10*). In the United States, the U.S. Department of Education, Office of Safe and Healthy Student, Readiness and Emergency Management for Schools Technical Assistance Center* and CDC’s Children’s Preparedness Unit^†^ have developed a suite of publications and tools to help schools and families prepare for, respond to, and recover from emergencies.

The findings in this report are subject to at least three limitations. First, SHPPS data are self-reported and thus are subject to bias. Second, SHPPS documentation states that the word “policy” refers to any law, rule, regulation, administrative order, or similar kind of mandate issued by the local school board or other local agency with authority over schools in districts; this might be interpreted differently by individual respondents. Finally, the binary response option (yes/no) does not indicate whether a school district has taken action on the policy in question.

During the past decade, more school districts have adopted policies requiring certain preparedness measures for schools. However, school districts have not met all of the target goals of the Healthy People 2020 PREP-5 objectives, indicating suboptimal preparedness planning in some localities. Findings from this report highlight the need for wider adoption of policies on family reunification, pandemic influenza and other infectious disease outbreak procedures, and provisions for students and staff members with special needs, particularly in rural areas. School district-specific information on school crisis preparedness planning and training might help identify and address disparities and critical gaps in preparedness and response policies and plans for children. Adoption of strong policies by school districts can promote the health and safety of faculty, staff members, children, and families and meet the Healthy People 2020 preparedness objectives [PREP-5] for safe school environments.

**Summary**

**What is already known about this topic?**

Healthy People 2020 includes objectives to improve school preparedness, response, and recovery plans in the event of a disaster.

**What is added by this report?**

Analyses of data found differences in trends by urbanicity in district requirements for crisis plans. In 2016, large districts (≥10,000 students) were significantly more likely than were small districts (≤4,999 students) to provide funding for or offer training on crisis preparedness for school faculty, staff members, and students’ families.

**What are the implications for public health practice?**

To meet Healthy People 2020 targets, increases are needed in district adoption and implementation of policies. Strengthening plans for infectious disease outbreaks, especially in rural districts, could help ensure that children and communities remain healthy and productive.

## Footnotes

* https://rems.ed.gov/.

† https://www.cdc.gov/childrenindisasters/before-during-after.html.
